# Cellular Distribution and Ultrastructural Changes in HaCaT Cells, Induced by Podophyllotoxin and Its Novel Fluorescent Derivative, Supported by the Molecular Docking Studies

**DOI:** 10.3390/ijms25115948

**Published:** 2024-05-29

**Authors:** Piotr Strus, Karol Sadowski, Julia Kostro, Andrzej Antoni Szczepankiewicz, Hanna Nieznańska, Magdalena Niedzielska, Andrei Zlobin, Pramukti Nawar Ra’idah, Zuzanna Molęda, Joanna Szawkało, Zbigniew Czarnocki, Cezary Wójcik, Łukasz Szeleszczuk, Izabela Młynarczuk-Biały

**Affiliations:** 1 Department of Histology and Embryology, Medical University of Warsaw, Chałubińskiego 5, 02-004 Warsaw, Poland; struspiotrek@gmail.com; 2 Students Scientific Group HESA, Department of Histology and Embryology, Medical University of Warsaw, Chałubinskiego 5, 02-004 Warsaw, Poland; karsad2001@wp.pl (K.S.);; 3 Laboratory of Electron Microscopy, Nencki Institute of Warsaw, Pasteura 3, 02-093 Warsaw, Polandh.nieznanska@nencki.edu.pl (H.N.); 4 Laboratory of Natural Products Chemistry, Faculty of Chemistry, University of Warsaw, Pasteura 1, 02-093 Warsaw, Poland; m.niedzielska3@student.uw.edu.pl (M.N.); a.zlobin@student.uw.edu.pl (A.Z.);; 5 Amgen Inc., Thousand Oaks, CA 91320, USA; wojcikc@ohsu.edu; 6 Department of Undergraduate Medical Education, OHSU School of Medicine, Portland, OR 97239, USA; 7 Department of Organic and Physical Chemistry, Faculty of Pharmacy, Medical University of Warsaw, Banacha 1, 02-093 Warsaw, Poland

**Keywords:** podophyllotoxin, HaCaT ultrastructure, podophyllotoxin with attached fluorescein, podophyllotoxin docking to tubulin, human keratinocytes, cell death

## Abstract

Podophyllotoxin (PPT) is an active pharmaceutical ingredient (API) with established antitumor potential. However, due to its systemic toxicity, its use is restricted to topical treatment of anogenital warts. Less toxic PPT derivatives (e.g., etoposide and teniposide) are used intravenously as anticancer agents. PPT has been exploited as a scaffold of new potential therapeutic agents; however, fewer studies have been conducted on the parent molecule than on its derivatives. We have undertaken a study of ultrastructural changes induced by PPT on HaCaT keratinocytes. We have also tracked the intracellular localization of PPT using its fluorescent derivative (PPT-FL). Moreover, we performed molecular docking of both PPT and PPT-FL to compare their affinity to various binding sites of tubulin. Using the Presto blue viability assay, we established working concentrations of PPT in HaCaT cells. Subsequently, we have used selected concentrations to determine PPT effects at the ultrastructural level. Dynamics of PPT distribution by confocal microscopy was performed using PPT-FL. Molecular docking calculations were conducted using Glide. PPT induces a time-dependent cytotoxic effect on HaCaT cells. Within 24 h, we observed the elongation of cytoplasmic processes, formation of cytoplasmic vacuoles, progressive ER stress, and shortening of the mitochondrial long axis. After 48 h, we noticed disintegration of the cell membrane, progressive vacuolization, apoptotic/necrotic vesicles, and a change in the cell nucleus’s appearance. PPT-FL was detected within HaCaT cells after ~10 min of incubation and remained within cells in the following measurements. Molecular docking confirmed the formation of a stable complex between tubulin and both PPT and PPT-FL. However, it was formed at different binding sites. PPT is highly toxic to normal human keratinocytes, even at low concentrations. It promptly enters the cells, probably via endocytosis. At lower concentrations, PPT causes disruptions in both ER and mitochondria, while at higher concentrations, it leads to massive vacuolization with subsequent cell death. The novel derivative of PPT, PPT-FL, forms a stable complex with tubulin, and therefore, it is a useful tracker of intracellular PPT binding and trafficking.

## 1. Introduction

Podophyllotoxin (PPT—(−)-podophyllotoxin) is an active pharmaceutical ingredient (API) that is clinically used as one of the few topical therapies to treat anogenital warts. Most cases of anogenital warts are caused by Human Papillomavirus (HPV) types 6 or 11 [1,2]. 

PPT was first isolated from *Podophyllum* spp. (family *Berberidaceae*). It is obtained mainly from the roots and the rhizomes of *Podophyllum peltatum* (American mayapple) and *Sinopodophyllum hexandrum* Royle (Barberry family) [3,4].

PPT has antimitotic activity and inhibits the polymerization of tubulin [5,6]. The exact mechanism underlying the antitumor effects of PPT remains unclear. Nevertheless, research indicates that PPT binds tubulin subunits, thus hindering its polymerization and formation of microtubules. PPT has a similar affinity and binding site to colchicine although PPT binds m—ore rapidly and reversibly than the irreversible binding of the latter [7]. PPT can also act as a competitive inhibitor of colchicine binding [8,9]. PPT binding leads to a G2/M arrest of the cell cycle by influencing the formation of the mitotic spindle [10]. Without the ability to divide, cells are arrested in the cell cycle and eventually die [11,12]. Due to its high toxicity, PPT can only be used topically—as either a 0.5% solution or a 0.15% cream. Even with topical use, the treatment schedule requires interruptions due to local and systemic side effects [2,13,14]. Local side effects include tenderness, erythema, erosion, and scarring of the skin in the place of application [2,15]. Less common are systemic side effects such as nausea, vomiting, diarrhea, abdominal pain, thrombocytopenia, leukopenia, abnormal liver function tests, sensory ataxia, and altered mental state [15,16,17]. 

PPT is frequently used as a scaffold for the synthesis of new derivatives that are created in the search of safer drugs with enhanced activity and less toxicity [10,18,19].

**Figure 1:**
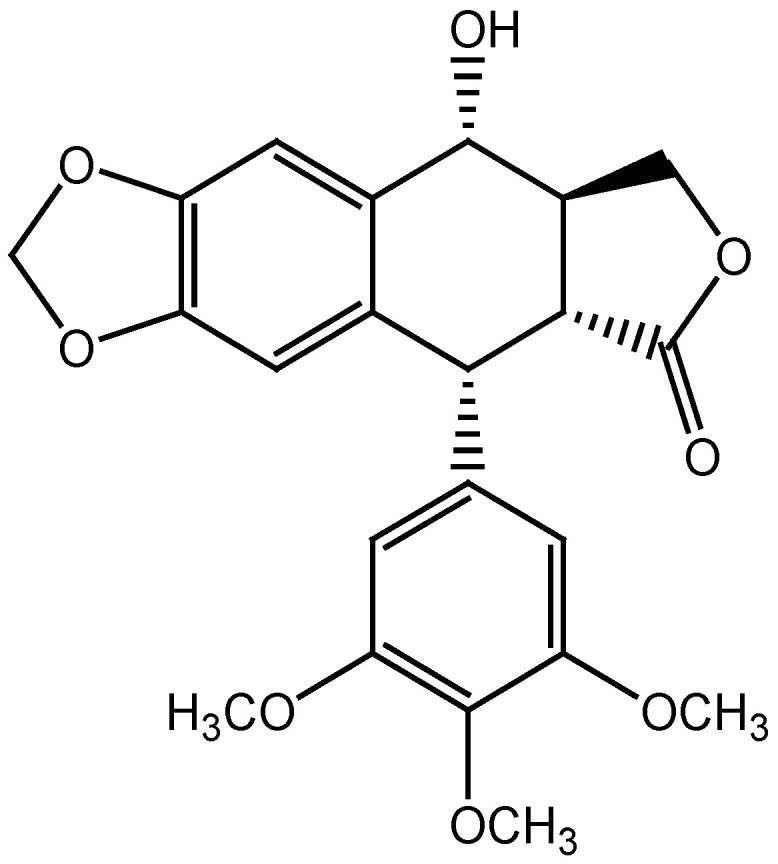
The structural formula of (−)-podophyllotoxin. Podophyllotoxin is an aryltetralin lignan lactone derived from *Podophyllum* spp. It consists of five rings with four chiral centers, one trans-lactone, and one aryl tetrahydronaphthalene skeleton with multiple modification sites.

PPT is formed by five rings with four chiral centers, a trans-lactone, and an aryl tetrahydrogenated naphthalene backbone (Figure 1). Due to challenging de novo-synthesis of PPT, modifications of naturally derived PPT are a common route to produce PPT analogs. In this process, the PPT structure is modified by adding functional groups or conjugation with another molecule. In this way, new PPT derivatives are synthesized [20,21,22,23]. The anticancer PPT derivatives used in the clinic include etoposide and teniposide [24,25]. They form part of protocols for the treatment of several forms of solid tumors, leukemias, and lymphomas. In contrast to the PPT mechanism of action, they inhibit DNA-topoisomerase II and thus stop the cell cycle during mainly S and G2 phases [3,26]. Other PPT derivatives also have immunosuppressive, antioxidant, antiviral, hypolipemic, and anti-inflammatory effects, thus showing great potential for possible future therapeutic use beyond oncology [27]. Many other PPT scaffold-based agents are being investigated (e.g., etopophos, etoposide phosphate, GL331, NK-611, TOP53, and NPF). They all have demonstrated possible anticancer activity [3,28]. In particular, our research group studied KL3—another PPT derivative that was found to be less toxic for non-cancerous cells and more specific to cancer cells than the parent PPT compound [15]. 

Since PPT is widely used in search of new active derivatives, we believe that a better understanding of its effects at the ultrastructural level is needed; this may give us new perspectives and ideas for pharmaceutical research related to aryltetralinlactone cyclolignans.

In this manuscript we analyzed the ultrastructural changes induced by PPT in HaCaT cells, and examined the cellular distribution of fluorescein-labeled PPT. We have then used molecular docking to compare the affinity of both PPT and PPT-FL towards tubulin.

## 2. Results

### 2.1. Properties of PPT-FL

According to the methodology described in Material and Methods, a new fluorescent derivative of PPT was synthesized (PPT-FL). It is a stable compound, well soluble in DMSO, and easily resolubilized in cell culture media. Next, we checked and compared the molecular docking of both PPT and PPT-FL to confirm a similar mechanism of action. Finally, both substances were tested in HaCaT keratinocytes in order to visualize their impact on cell viability and cellular distribution. PPT-FL at a concentration of 100 µM (1 µL/mL) gave bright intra-cellular fluorescence with a speckled pattern that was stable for 24 h (see results of cell-based assays).

### 2.2. Molecular Docking and MM/GBSA Calculations

**Figure 2:**
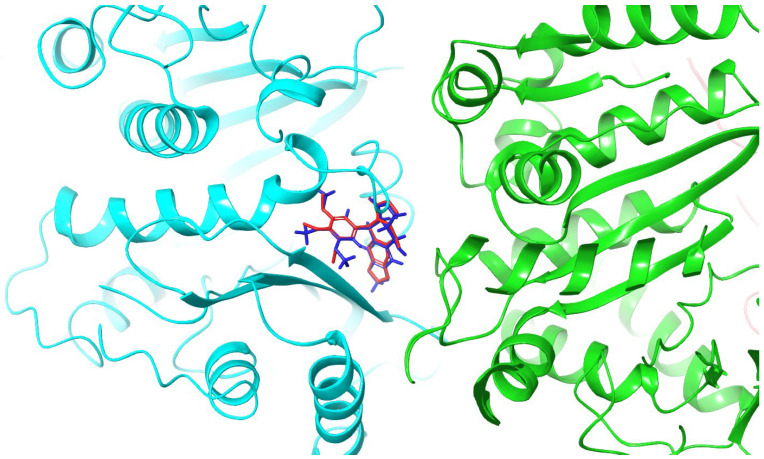
Comparison of the conformation and location of the PPT in the active site of tubulin (1SA1) from the crystal structure (red) and molecular docking studies (blue).

As stated in the Materials and Methods Section 4, a careful review of the previously deposited crystal structures of tubulin bound to various ligands allowed us to choose 4 structures representing 4 different binding sites of this protein. The 1SA1 site presented the structure of the complex between PPT and tubulin; therefore, we have first docked the studied ligand to 1SA1 to check the accuracy of the molecular docking in predicting the structure of the complex and the conformation of PPT at the active site (Figure 2). This approach is known as re-docking. 

The accuracy of the molecular docking in predicting the geometry of the PPT at the active site of tubulin is evidenced by the alignment of theoretically predicted and experimental structures. The rings of the PPT molecule overlap almost completely. Minor differences are seen between the angles formed by the methoxy groups.

We have also performed the molecular docking and MM/GBSA using PPT-FL instead of the parent PPT molecule (Table 1). Despite using grids of a size sufficient enough to fully accommodate PPT-FL, this ligand did not fit into the active sites of 1SA1, 4O2B, and 3HKD, which was mostly caused by steric hindrance. This suggests that PPT-FL most likely binds at another active site, which is different from the site of PPT binding. Indeed, PPT-FL formed a complex with tubulin binding at the “epothilone binding site”, presented in 1TVK. This complex is stabilized by three hydrogen bonds formed between the PPT-FL and tubulin, involving residues THR274, ARG282, and GLY360.

**Figure 3:**
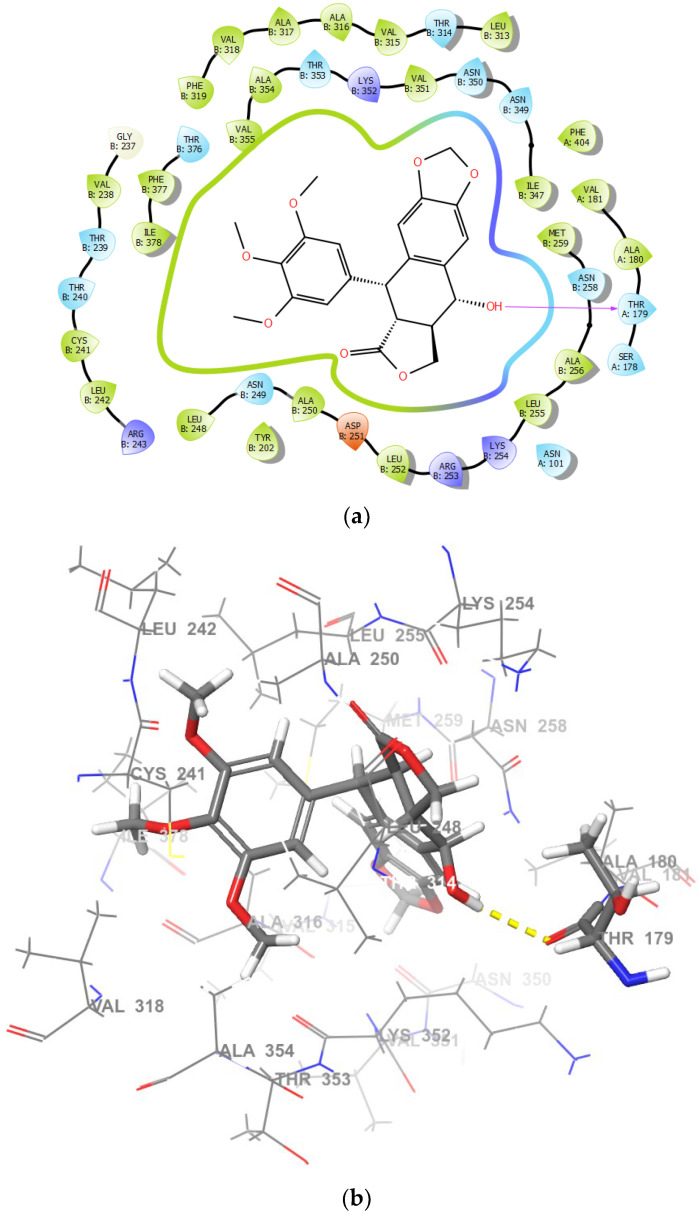
(**a**): Protein-ligand interactions between PPT and tubulin (1SA1). The violet line represents the H-bond. (**b**): Conformation of PPT in the active site of tubulin (1SA1). The yellow dashed line represents the H-bond.

**Figure 4:**
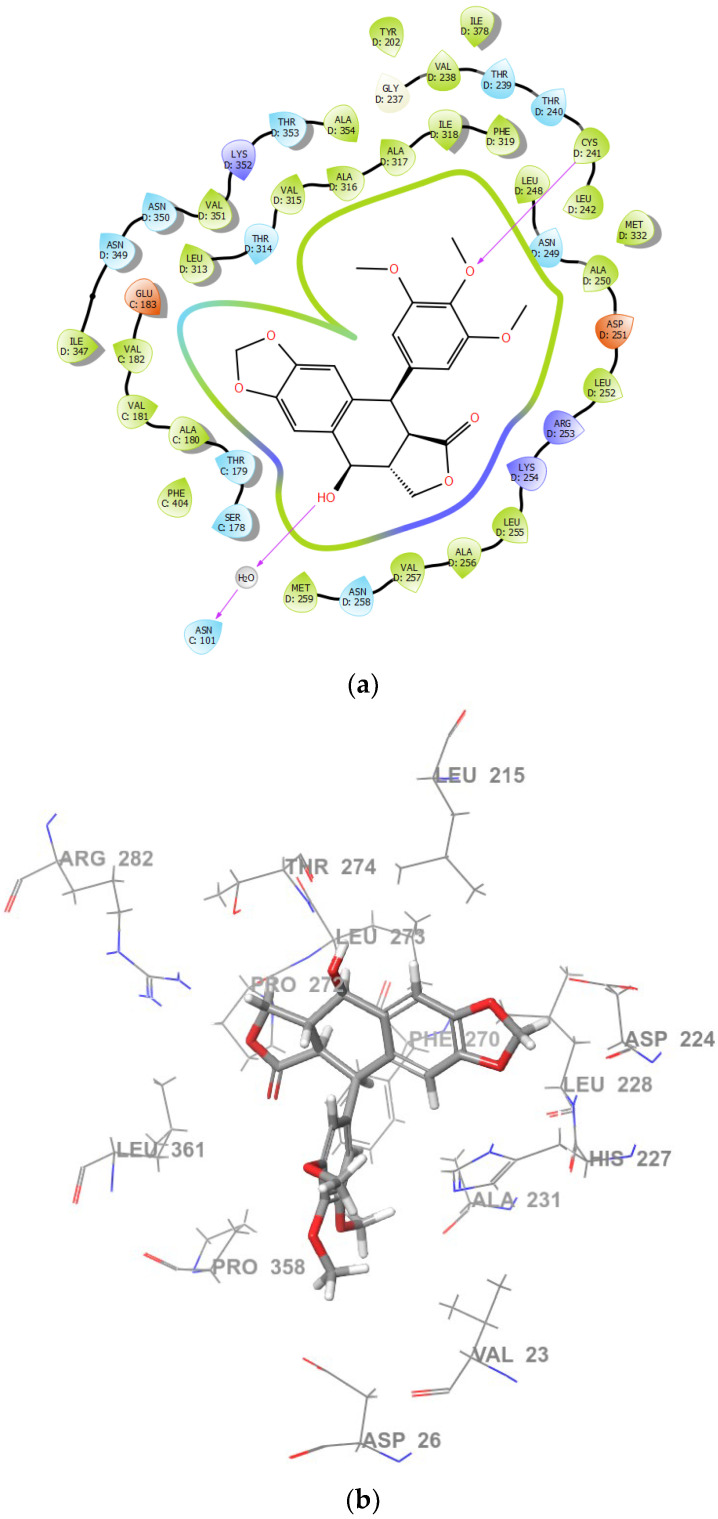

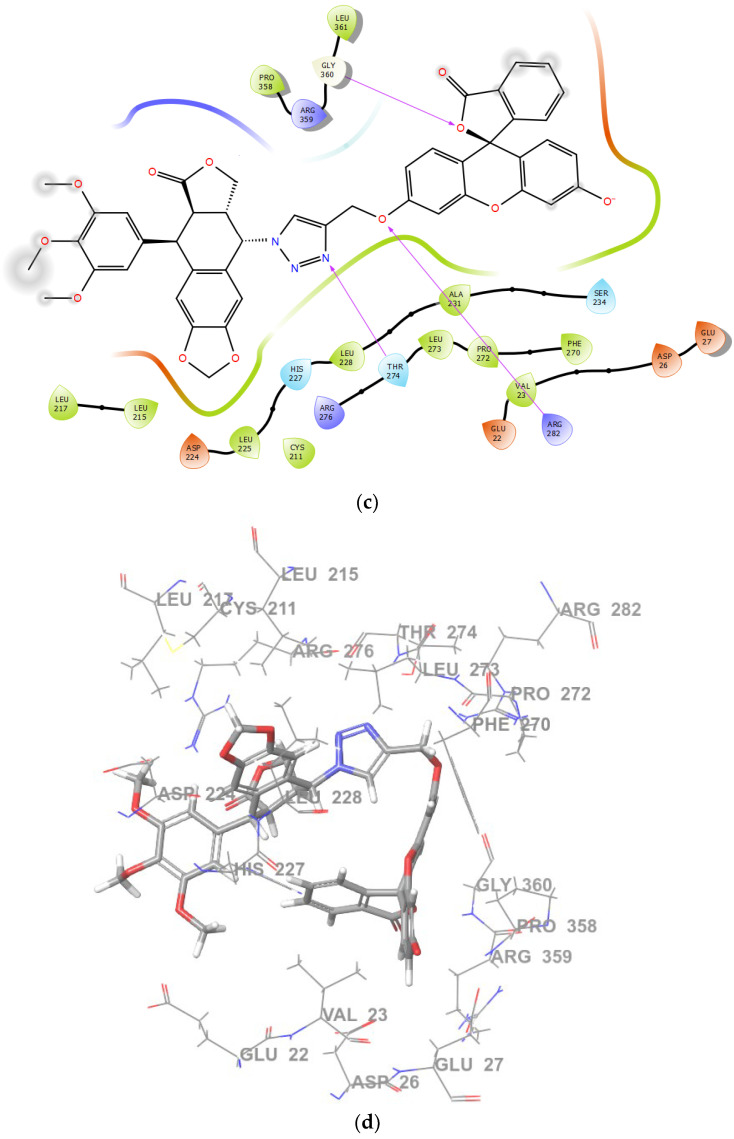

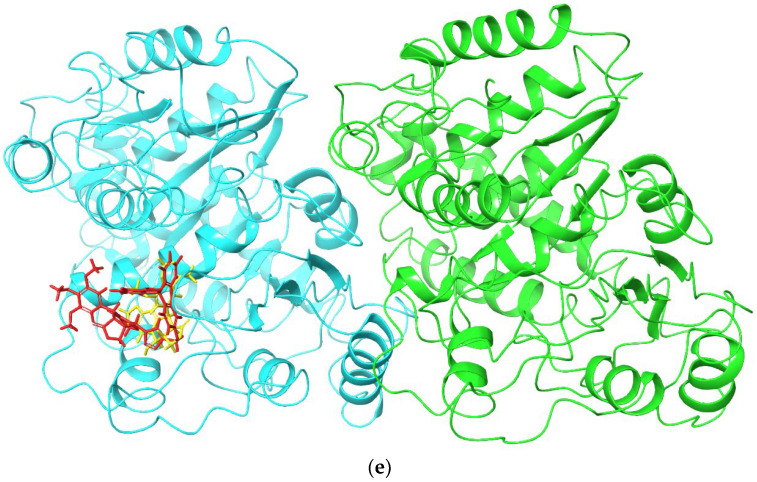
(**a**): Protein-ligand interactions between PPT and tubulin (1TVK). Violet lines represent the H-bonds. (**b**): Conformation of PPT in the active site of tubulin (1TVK). (**c**): Protein-ligand interactions between PPT-FL and tubulin (1TVK). Violet lines represent the H-bonds. (**d**): Conformation of PPT-FL in the active site of tubulin (1TVK). (**e**): Location of PPT (yellow) and PPT-FL (red) in the active site of tubulin (1TVK).

Analysis of the binding between PPT and tubulin revealed that the complex with the highest ligand affinity (−8.11491 kcal/mol and −61.9107 kcal/mol for GScore and MM/GBA, respectively; Figure 3) is obtained when the PPT is docked at the site known as the “PPT binding site” what is corroborated by the experimental data. We have also observed PPT binding at two other sites (1TVK, 4O2B). However, it had significantly lower affinities [X and Y, respectively]. In two complexes, formed with 1SA1 and 4O2B binding sites, respectively, the structure was stabilized by hydrogen bond formation between the hydroxyl group of PPT and Thr179 and Thr274 in case of 1SA1 and 4O2B binding sites, respectively. Interestingly, a complex of PPT bound to the 1TVK binding site (Figure 4) was characterized by lower affinity according to the Glide GScore and MM/GBSA values despite being stabilized by two H-bonds, formed with Asn101 and Cys24.

### 2.3. Cell-Based Assays

#### 2.3.1. Viability Assay

**Figure 5:**
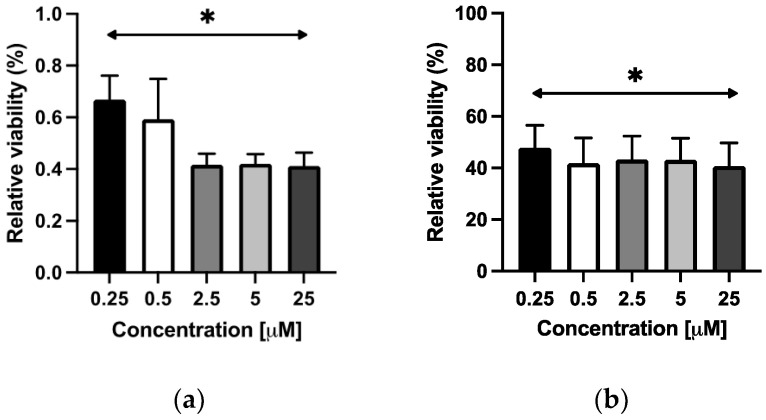
The viability of HaCaT cells is presented as a ratio percentage compared to the control group after incubation with increasing concentrations of PPT at 24 h (**a**) and 48 h (**b**). Stars: *p* < 0.05 at one-way ANOVA test. Compared to the control groups, for which the viability was 100% and is not shown in the figure, a statistically significant reduction in the viability of HaCaT cells was observed for each concentration. For concentrations up to 2.5 µM at 24 h, the viability-reduction effect was dose-dependent. Above this concentration, the graph flattens out, probably due to the saturation of the effects and the lack of linearity of the viability reduction effect for higher doses.

HaCaT cells were treated with PPT at concentrations ranging from 0.25 to 25 μM for either 24 h or 48 h. After 24 h at 0.25 μM and 0.5 μM, PPT caused ~40% reduction in viability, leaving ~60% of live cells in comparison to controls treated with PPT solvent alone. After 48 h incubation, PPT reduced the HaCaT viability below 50% at both concentrations. This effect was dose-dependent at 0.25 μM and 0.5 μM. Above 0.5 μM, increasing concentrations demonstrated a plateau effect without further increase in cytotoxicity. Each of these concentrations reduced HaCaT viability to about 40% as early as after 24 h (Figure 5a,b). Ultrastructural changes were more pronounced with 0.5 μM treatment in comparison with 0.25 μM treatment for the same incubation time and for the longer incubation time versus the shorter incubation time for each given concentration. We calculated the IC_50_ value at both time points, and for PPT, it was 1 µM after 24 h and 0.4 µM after 48 h. In addition, the IC_50_ value for PPT-FL was 0.4 µM after 48 h of incubation.

#### 2.3.2. Intracellular Distribution of Fluorescent PPT Derivative (PPT-FL)

**Figure 6:**
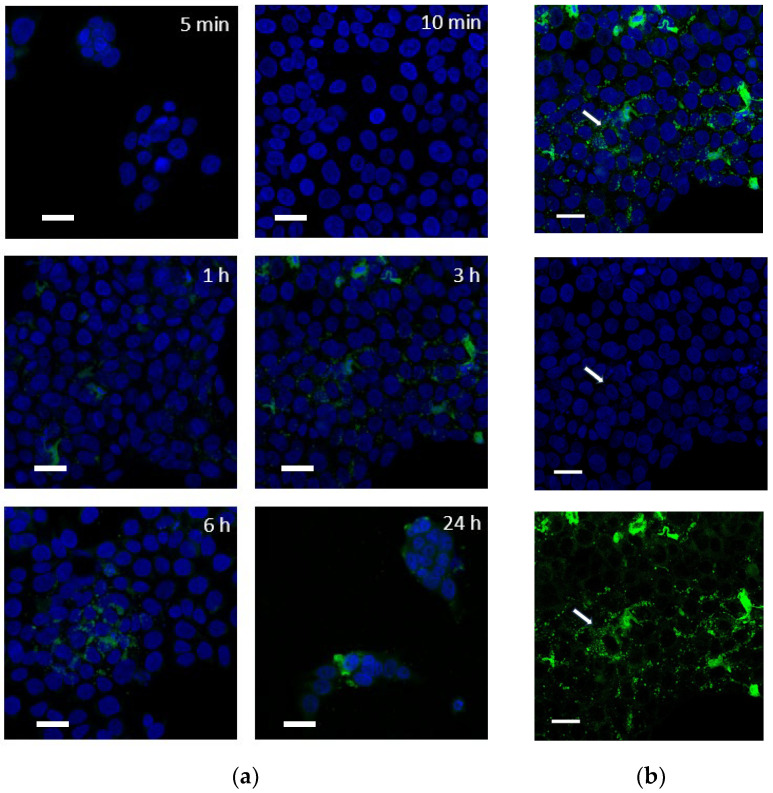
Time-dependent distribution of PPT-FL in HaCaT cells. Ha-CaT cells were incubated with PPT-FL (green), and nuclei were stained with HOECHST (blue). PPT-FL concentration 1 µL/mL—corresponding to 100 µM concentration of PPT. (**a**) Time-dependent analysis of PPT-FL distribution in HaCaT cells at 5 min, 10 min, 1 h, 3 h, 6 h and 24 h incubation with PPT-FL. (**b**)—Distribution of PPT-FL after 8 h of incubation—upper panel -merge, middle panel: HOECHST, lower panel: PPT-FL; speckled distribution of PPT-FL labeled structures in the cytoplasm of HaCaT cells. The white arrow shows the same cell in all three images. Scalebar: for (**a**,**b**): 20 µm.

After docking experiments conformed binding to tubulin (albeit at different sites) of both PPT and PPT-FL, we used confocal microscopy to study intracellular localization of PPT-FL labeled structures in vivo. We have shown that PPT-FL enters HaCaT cells in a time-dependent manner. The bright intracellular fluorescence signal can be observed starting from 1 h after its addition to the cell culture media (Figure 6, middle and lower panel). Up to 10 min after PPT-FT addition, no detectable fluorescent signal was observed within cells. PPT-FL labeled structures appear to be intracytoplasmic inclusions or vesicles with speckled patterns in the cytoplasm of HaCaT cells. Formation of such inclusions appears to be a continuous process as their abundance and staining intensity increase with prolonged incubation times. After 24 h of incubation, a bright patched cytoplasmic signal was also visible. This suggests that PPT-FL is neither degraded nor excluded from the treated cells (Figure 6—lower right panel, 24 h).

#### 2.3.3. Ultrastructural Analysis

**Figure 7:**
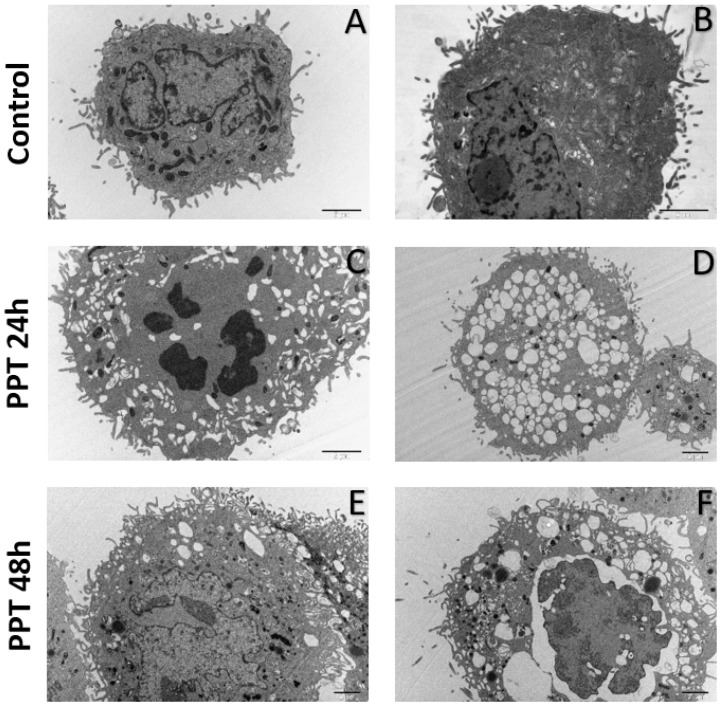
Ultrastructural changes of HaCaT cells treated with PTX for 24 h (**C**,**D**) and 48 h (**E**,**F**). In comparison to the control cells (**A**,**B**), PTX induces dilation of ER, suggesting presence of ER stress. All changes are more pronounced after a longer time of incubation. Scalebar 1 µm.

To further characterize intracytoplasmic changes caused by PPT in HaCaT cells, we used transmission electron microscopy. Control HaCaT cells are displayed in Figure 7A,B. They contain a centrally located normally appearing cell nucleus with abundant heterochromatin, in one case with a nucleolus visible (B). Various cytoplasmic organelles are localized in the peri-nucleolar region, with normally appearing mitochondria and ER. Cell membranes are continuous, and cellular protrusions are visible.

After treatment with PPT for 24 h, peripheral nuclear chromatin condensation is visible, while cytoplasm remains homogenous, with fewer mitochondria in comparison to controls and with intensively dilated ER. ER dilation is more pronounced in the peripheral region of the cytoplasm than in the perinuclear region (Figure 7C). The intensive vacuolization of ER is more advanced in Figure 7D—where the apical region of the cell is visualized.

Cells incubated for 48 h with PPT revealed more advanced vacuolization with sequestration of the cell nucleus (Figure 7E,F) and further dilation of ER cisternae in comparison with the shorter incubation time.

**Figure 8:**
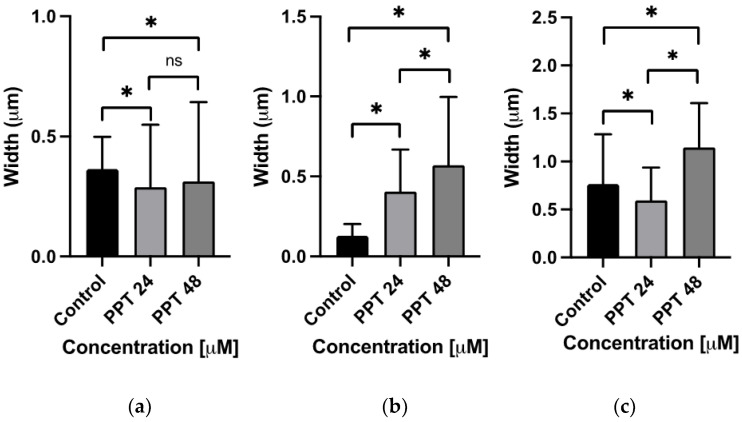
Comparison of mitochondrial length (**a**), width of endoplasmic reticulum cisternae (**b**), and length of cellular protrusions (**c**) in HaCaT cells treated with PPT at 10 μM for 24 h and 48 h as compared with controls. Stars: *p* < 0.05 at one-way ANOVA test, ns: not significant.

Morphometric analysis demonstrated that incubation of HaCaT cells with 10 μM PPT for 24 h led to significant morphological changes in ER, cellular processes, and mitochondria (Figure 8). The 24 h incubation with 10 μM PPT causes mitochondria shortening; however, after 48 h, mitochondria appear similar to the controls. After both 24 h and 48 h incubation, there appears to be no change in mitochondrial combs with similar width, length, and electron density. Nevertheless, despite the shortening of mitochondria, PPT does not appear to disrupt the integrity of either outer or inner mitochondrial membranes, which remain continuous and well delimited from the cytoplasm. At the same time, PPT causes progressive widening of ER cisternae, which is universally considered a hallmark of ER stress. Finally, PPT appears to induce an initial shortening of cytoplasmic processes. After an extended 48 h incubation, this shortening appears reversed, with cytoplasmic processes becoming more elongated than in control cells. We did not observe any changes in the ultrastructure of the cell nucleus, such as, e.g., chromatin condensation, pyknosis, or cell nucleus fragmentation after either 24 h or 48 h in most interphase cells, with exception of cells with massive vacuolization and disintegration of membranes where signs of apoptosis were detected (Figure 7F). In summary, 24 h treatment with 10 μM PPT-induced constant ER stress temporally shortens mitochondria as well as elongation of cytoplasmic processes.

Mitochondria showed a tendency towards reduction in mitochondrial length at both time points. The width of the ER cisternae increased in a time-dependent manner, achieving statistically significant ER widening at both time points. Cytoplasmic protrusions were shortened after 24 h, but after 28 h they were longer than in the control group.

## 3. Discussion

Despite advances in chemotherapy and immunotherapy, cancer remains one of the top killers, especially in developed countries, second only to cardiovascular disease. Its incidence and prevalence have increased worldwide. Therefore, the need arises for new therapeutic agents with greater precision and better safety than those currently available. This is especially true because as the disease progresses, cancer cells become drug-resistant.

The drug development process is long, arduous, and expensive. However, it can be accelerated and its costs reduced by using proven scaffold molecules with good bioavailability as a starting point for drug design. 

PPT is an example of such a scaffold molecule, which has been used to obtain several derivatives [3,4]. The initial challenge was to improve PPT solubility in water to enable the systemic use of PPT derivatives [29,30]. Indeed, PPT derivatives such as etoposide and teniposide are water soluble and, therefore are used as part of systemic chemotherapy regimens against multiple cancer types [30,31]. Their antineoplastic activity is mediated via the inhibition of DNA-topoisomerase II, leading to cell cycle block and apoptosis [32]. Derivation of etoposide and teniposide was possible because of the thorough study of PPT itself [3]. We believe that additional in-depth PPT investigation will facilitate the development of further PPT derivatives with novel therapeutic applications.

The design and synthesis of the new fluorescent PPT derivative (PPT-FL) was undertaken in order to better understand the intracellular localization of PPT within normal human keratinocytes. PPT-FL turned out to be a stable compound, enabling its tracking for at least 24 h in vivo. Furthermore, this derivative was found to be stable after fixation with ethanol and did not separate from cells during washing or HOECHST staining, likely reflecting strong binding to cellular structures. As far as we know, this is the first report on the synthesis and in vivo use of a PPT fluorescein-labeled derivative.

Molecular docking analysis has confirmed that both native PPT and the PPT-FL derivative could bind to tubulin. However, it appeared that PPT-FL did not fit into the same site as PPT. Instead, PPT-FL formed a complex at another active site of the tubulin dimer, known as the “epothilone binding site” or “taxane binding pocket” [33]. Our findings suggest that it is necessary to confirm the molecular fit of each new compound derived from a known parent molecule since spatial modification(s) can affect the docking position at the target protein complex. In addition, the inclusion of compound 2 may result in a new biological activity of PPT-FL.

Following a rapid uptake occurring within 60 min, likely via endocytosis, PPT-FL remains stable in vivo for at least 24 h. This contrasts with the short half-life of the parent PTT (1–4.5 h) and reported studies on the duration of PPT impact on the cytoskeleton [34]. PPT-FL demonstrates that it is possible to easily synthesize fluorescent PPT derivatives, which can be used to track PPT and its other derivatives within cells. This holds a promise for advancing our knowledge and potential therapeutic applications in the field of oncology [35].

Cell-based assays using HaCaT keratinocytes have confirmed the cytostatic/cytotoxic effects of PPT. This effect was dose-dependent until a plateau was reached without any further reduction of viability, with subsequently increasing doses suggesting a saturation of intracellular mechanism mediating this effect. Similar effects of PPT itself and its various derivatives have been reported using different cell culture systems [15,18]. It is important to note that even in clinical trials, PPT causes death and cytostatic effects in human keratinocytes [36].

We have focused our analysis on interphase cells, which were prevalent in our cell culture system.

Consistent with previous studies [15], PPT appeared to be highly cytotoxic. Even at low concentrations, it causes over 50% of the cells to die within 24 h. However, this effect is saturable as further increases in PPT concentrations do not result in increasing rates of cell death. Observed minor changes in mitochondrial morphology, suggests that PPT does not affect significantly mitochondrial structure or function. Initial shrinking in size followed by its recovery after a longer incubation suggests an adaptive mechanism, which requires further studies [15]. 

In contrast, following PPT incubation, the ER underwent significant changes, manifested as dilation of the ER cisternae, which was observed at 24 h and progressed further at 48 h. Vacuole formation likely represented extreme dilation of ER cisternae. Those morphological changes were consistent with ER stress, pointing towards the possible involvement of this mechanism in the initiation of programmed cell death in those cells, similar to previous reports [15,37,38]. ER stress is characterized by the accumulation of unfolded and/or misfolded proteins within the ER lumen, a process that can be triggered by the expression of abnormal proteins, proteasome inhibition, nutrient deficiency, hypoxia, calcium depletion, and other conditions. ER stress triggers a complex cellular reaction known as Unfolded Protein Response (UPR), which aims to restore ER homeostasis [39]. However, if UPR fails and/or ER stress is prolonged, it eventually leads to caspase activation and initiation of programmed cell death [40].

PPT caused an initial decrease in the length of cell protrusions (podia) after 24 h, which was followed by a relative increase after 48 h (albeit still below its original length). This observation indicates that PPT initially affects cytoskeletal components, resulting in a shortening of cellular protrusions. Further incubation likely initiates a compensatory response, which promotes protrusion regrowth, consistent with the known effects of PTT on filament dynamics [41,42]. This intriguing biphasic response suggests a complex interaction between PPT and cellular morphology that warrants further investigation.

This study provides valuable information on the effects of PPT on ultrastructure and viability of HaCaT cells. The observed cytotoxicity, morphological changes, and rapid cellular uptake of PPT highlight its potential as a backbone for new derivatives with better bioavailability and improved adverse effect profile. Our study has a few limitations. First, we have used only one cell line cultured in vitro, and extrapolation of these results to other cell types as well as to entire organisms should be approached with caution. Additionally, the ultrastructural changes observed in this study should be further correlated with mechanistic studies. More research is needed to investigate the effectiveness, safety, and mechanisms of action of PPT and its derivatives in different types of cancer cells using various in vitro and in vivo models. Fluorescent labeling of PPT and its derivatives can prove a valuable tool in such an endeavor.

## 4. Materials and Methods

### 4.1. Chemicals

PPT was purchased from Sigma-Aldrich (Thermo Fisher Scientific, Inc., Waltham, MA, USA). It was dissolved in DMSO at 100 mM stock solution and kept at −20 °C until use.

### 4.2. Chemical Synthesis

A previously unknown fluorescently labeled PPT derivative, which can be used in numerous studies, was synthesized in a simple and efficient way (PPT-FL). First, building blocks for a “click” reaction were synthesized. Subsequently, PPT underwent an SN2 reaction, in which the hydroxyl group was exchanged with an excellent yield to an azide, giving compound **1**. In turn, fluorescein was transformed into an alkyne derivative (**2**) in one step, using an etherification reaction with propargyl bromide. 

**Scheme 1:**
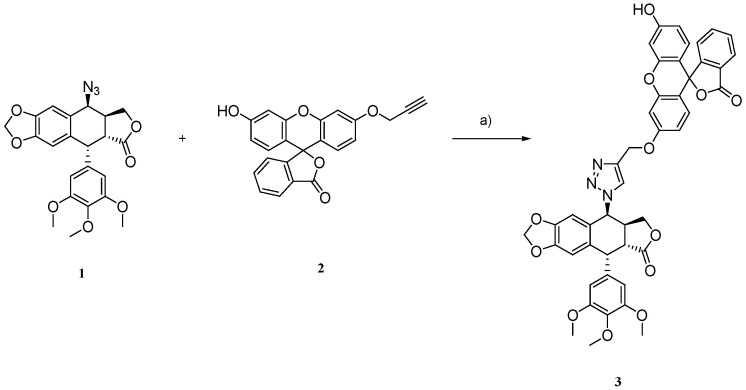
Synthesis of fluorescent-labeled podophyllotoxin. The following numbers in the diagram correspond to chemical formulas: (**1**) podophyllotoxin-derived azide; (**2**) fluorescein-derived alkyne; (**3**) fluorescent-labeled podophyllotoxin (PPT-FL); (a) CuSO_4_∙5H_2_O, sodium ascorbate, THF/H_2_O, rt, 35%.

The fluorescein-labelled podophyllotoxin 3 (PPT-FL) was synthesized via a copper-catalyzed cycloaddition reaction between a PPT-derived azide (**1**) and an fluorescein-derived alkyne (**2**) (Scheme 1). The reaction was performed with acceptable yield, without generating side-products. PPT-FL isolation was straightforward and allowed us to obtain a pure compound, suitable for analytical characterization and biological testing.

This method for synthesizing a fluorescent PPT derivative is simple, affordable, and can be performed even in non-specialized laboratories.

In particular, according to Scheme 1, PPT-derived azide **1** and fluorescein-derived alkyne **2** were prepared according to the procedures previously described [43,44]. A PPT-derived azide (0.05 mmol) and a fluorescein-derived alkyne (0.05 mmol) were dissolved in THF (4 mL). CuSO_4_∙5H_2_O (0.15 eq). Then, sodium ascorbate (0.45 eq) dissolved in water (1 mL) was added to the solution, and the reaction mixture was stirred for 24 h. Subsequently, THF was removed under reduced pressure, brine (10 mL) was added, and the water solution was extracted three times with chloroform. The combined organic phases were washed with brine, dried over anhydrous MgSO_4_, and filtered. The solvent was removed under reduced pressure, and the product was purified using column chromatography (silica gel, 2% methanol in chloroform). PPT-FL was obtained as a colorless oil with a 35% yield.

The ^1^H NMR and ^13^C NMR spectra were recorded on a Bruker AVANCE apparatus. High-resolution mass spectra were recorded on the Shimadzu LC 9030 apparatus (Shimadzu, Kyoto, Japan) using the TOF MS ES+ method.

^1^H NMR (300 MHz, CDCl_3_), δ (ppm): 7.99 (d, J = 7.2 Hz, 1H, H_fluoresc_), 7.63 (m, 2H, 2H_fluoresc_), 7.40 (d, J = 1.8 Hz, 1H, H_triazole_), 7.14 (m, 1H, H_fluoresc_), 6.82 (d, J = 2.1 Hz, 1H, H_fluoresc_), 6.73 (d, J = 2.1 Hz, 1H, H_fluoresc_), 6.68–6.52 (m, 4H, 2H_fluoresc_, H-5, H-8), 6.32 (s, 2H, H-2′, H-6′), 6.10 (d, J = 3.9 Hz, 1H, H-4), 5.97 (d, J = 13.2 Hz, 2H, O-CH_2_-O), 5.14 (s, 2H, triazoleCH_2_), 4.75 (d, J = 4.8 Hz, 1H, H-1), 4.37 (m, 1H, H-11), 3.81 (s, 3H, OCH3), 3.75 (s, 7H, H-11, 2OCH_3_), 3.31–3.10 (m, 2H, H-2, H-3), 1.77 (br. S, 1H, OH).

^13^C NMR (75 MHz, CDCl_3_), δ (ppm): 173.4, 170.0, 159.8, 152.9, 152.6, 149.6, 148.3, 143.6, 137.7, 135.4, 134.4, 133.4, 129.9, 129.3, 126.8, 125.1, 124.5, 123.8, 112.8, 112.1, 110.7, 108.9, 108.4, 103.3, 102.2, 77.4, 62.0, 60.9, 59.1, 56.5, 43.8, 41.7, 37.2. 

HR MS ES(+) (*m*/*z*) calculated for C_45_H_36_N_3_O_12_ ([M + H]^+^) 810.2294, found 810.2297.

### 4.3. Molecular Docking

Schrӧdinger Maestro 12.8. version (Schrӧdinger, LLC, New York, NY, USA, 2023) was used for molecular docking and Molecular Mechanics/Generalized Born Surface Area (MM/GBSA) computations.

#### 4.3.1. Structural Modeling

Three-dimensional crystal structures of tubulin bound to the inhibitors of tubulin polymerization were retrieved from the RCBS PDB database [45]. A careful search of the RCBS database revealed four binding sites at which the ligands were located. For each of the sites, we have chosen the structure of the ligand: tubulin complex with the best resolution. Detailed information on the selected binding sites is presented in Table 2.

The Protein Preparation Wizard was used to prepare the structural models before docking in order to eliminate undesired metal ions and water molecules. In addition, this allows the formation of disulfide bonds, assignment of proper bond orders, adjustment of ionization states, simplification of multimeric complexes, and correction of the orientation of misoriented groups. The protein structure was further modified by adding hydrogen atoms, and standard protonation states at pH 7.0 were applied. Geometrically stable structural models were then produced by optimizing and minimizing the preprocessed models. After that, molecular docking was performed using the generated protein structure models.

#### 4.3.2. Active Site Identification and Grid Generation

The grid center in each instance was the mass center of the co-crystallized ligand. The grid size of the cubic search box was chosen to a size that would allow the ligands to fit within completely. Using the OPLS 2005 force field, receptor grids were created using the default values for the charge cutoff (0.25) and van der Waals scaling factor (1.00).

#### 4.3.3. Ligands Preparation

Using LigPrep from the Schrödinger Suite, the investigated ligands were made ready for docking: protonation states were produced at pH 7.4 ± 2.0 while maintaining the chiralities. Utilizing the OPLS 2005 forcefield, the geometry optimization was performed. The default values for LigPrep’s other options were maintained.

#### 4.3.4. Glide XP-Ligand Docking

The Grid-based Ligand Docking with Energetics (Glide) module and the extra precision (XP) approach were used to accomplish flexible protein-ligand docking. The first Glide docking stage’s preset 20 postures were kept during the docking process. The glide score, or GScore, was determined as:

GScore = 0.065 × vdW + 0.130 × Coul + Lipo + Hbond + Metal + BuryP + RotB + Site

where vdW: van der Waals energy; Coul: Coulomb energy; Lipo: Lipophilic term; Hbond: Hydrogen-bonding; Metal: Metal-binding term; BuryP: Buried Polar groups’ penalty; RotB: Penalty for rotatable bonds that have been frozen; Site: active site polar interactions. 

#### 4.3.5. Evaluation of Molecular Docking

Re-docking and cross-docking procedures were performed to assess molecular docking accuracy. Re-docking is the capacity to replicate the co-crystallized binding geometry and orientation of the related ligand in a rigid macromolecule form. Cross-docking is the process of comparing distinct ligand structures isolated from several crystal structure data of the same protein to a single rigid protein model structure. In all situations, success is measured as the root mean squared deviation (RMSD) between the docked position and the corresponding crystal conformer. RMSD levels below 2.0 Å are regarded desirable, although values near zero are optimal.

Glide’s cross-docking script, xglide.py, was used to perform re-docking and cross-docking using the seven available crystal structures of tubulin stathmin-like domain complexes, refcodes 1SA1, 3HKC, 3HKD, 3HKE, 3N2G, 3N2K, 3UT5. Ligands from each structure were docked to the enzyme from each complex, using the Glide extra precision approach. The quality of fit was evaluated on the basis of ligand RMSD values with the following ranges: RMSD ≤ 1.0 Å for good pose, 1 Å < RMSD ≤ 2 Å for close pose, 2 Å < RMSD ≤ 3 Å for pose with errors and RMSD > 3 Å for bad pose. The calculated results are presented in Table 3. 

As expected, the lowest RMSD values were obtained for the re-docking. The lack of poses with errors and a significant majority of good poses indicate the good accuracy of the Glide docking approach and its applicability to perform the molecular docking for the studied compounds.

#### 4.3.6. MM-GBSA Calculations

To evaluate ligand binding affinities in silico, binding free energy (ΔG_bind_) values were calculated. The Molecular Mechanics/Generalized Born Surface Area (MM/GBSA) rescoring approach was utilized to accurately calculate free energies of binding. The Prime MM/GBSA module was employed for this purpose.

For the first ligand-docked postures with the best scoring functions, MM/GBSA rescoring was conducted. Using the VSGB solvent model and the OPLS 2005 force field, the free energy changes that occur during protein-ligand interactions were computed.

The values of binding free energy were computed using the subsequent formula:

MM/GBSA ΔG_bind_ = G_complex(optimized)_ − (G_protein(optimized)_ + G_peptide(optimized)_)

Free energy of each state, i.e., of complex, protein, and peptide, was estimated by accounting for molecular mechanics energies, solvation energies, and entropic terms as follows:

G = G_int_ + G_Coulomb_ + G_vdW_ + G_GB_ + G_lipo_ − TS

where G_int_, G_Coulomb_, and G_vdW_ are standard MM energy terms for bond (covalent, angle, and dihedral), Coulomb (electrostatic) and van der Waals interactions, G_GB_ and G_lipo_ are polar and non-polar (lipophilic) contributions to the solvation free energies, while T is an absolute temperature and S is an entropy value. Polar contribution (G_GB_) was calculated using the Generalized Born model, while non-polar contribution (G_lipo_) was estimated based on the solvent-accessible surface area (SASA).

### 4.4. Cell-Based Methods

#### 4.4.1. Cell Culture

HaCaT cells (ATCC, Manassas, VA, USA), not exceeding 6 months of cell culture, were cultured in DMEM (Gibco; Thermo Fisher Scientific, Inc., Waltham, MA, USA) containing stable glutamine and supplemented with 10% FBS and a standard antibiotic antimycotic solution (Gibco). Cells were grown in a humidified atmosphere at 37 °C and passaged every three days with a standard Trypsin-EDTA solution (Gibco). Cells were tested for mycoplasma contamination by staining with HOECHST (Sigma-Aldrich, Saint Louis, MO, USA) and followed by confocal microscopy analysis using a Leica-SP4 (Leica, Wetzlar, Germany) microscope. 

#### 4.4.2. Viability Assays

To determine the cytotoxic/cytostatic effects of compounds involved in this study, we performed a Presto Blue assay (Thermo Fisher) as described in [46]. For this assay, cells were seeded in 96 well plates (Nest Biotechnology, Wuxi, China) at the density of 10^4^ cells/well and treated in quadruplicates with increasing concentrations of PPT for 24 h or 48 h. After incubation, the Presto blue assay was performed according to the manufacturer’s instructions. The absorbance of a particular probe was read in a Fluostar plate reader at 550 nM (BMG Labtech, Ortenberg, Germany). Cell viability was expressed as a percentage of the control (vehicle-treated) cells. All results in the study were based on at least three experiments. The experimental compounds were dissolved in DMSO, which could affect cell viability. Therefore, an initial experiment was performed to investigate the toxicity of DMSO. We tested DMSO in increasing concentrations, ranging from 0.1% to 5% for 48 h on HaCaT cells. The maximum concentration of DMSO, which did not decrease cell viability, was found to be 0.5%. We, therefore used 0.1% as the maximal DMSO concentration as a solvent, which constituted the control for cell-based assays. We determined viability curves from the viability test data, and from these curves, we calculated the concentration causing 50% of the effect compared to the controls, hereinafter referred to as IC_50_. The IC_50_ was estimated for both PPT and PPT-FL.

#### 4.4.3. Confocal Microscopy

Distribution of fluorescence associated with PPT-FL in HaCaT cells was performed using confocal microscopy (Zeiss LSM 510, Oberkochen, Germany). After incubation with PPT-FL in culture media—cells were washed 2 × 5 min with PBS, fixed for 2 min in cold, buffered 70% ethanol, washed twice with PBS, soaked in 0.9% NaCl, stained with Hoechst (Sigma Aldrich, Saint Louis, MO, USA) according to manufacturer’s procedure, finally mounted in Vecta Shield mounting medium (Vector Laboratories, Zürich, Switzerland). An argon laser (488 nm) was used for the excitation of PTX-FL, and diode 405 nm was used for the excitation of Hoechst. Images were processed using the software package ZEN 2008.

#### 4.4.4. Transmission Electron Microscopy (TEM)

HaCaT cells were plated on tissue culture dishes, treated with PPT for 24 h or 48 h, then washed in PBS, trypsinized, harvested, and centrifuged. The cell pellet was fixed in 2% glutaraldehyde in 0.1 M sodium cacodylate (NaCac) buffer (pH 7.4), postfixed in 2% osmium tetroxide in NaCac, stained en bloc with 2% uranyl acetate, dehydrated with a graded ethanol series, and embedded in epon resin (all from Sigma Aldrich). Ultrathin sections were cut with a diamond knife on a Leica EM UC6 ultramicrotome (Leica Microsystems, Bannockburn, IL, USA), collected on copper grids, and stained with uranyl acetate and lead citrate. Cells were observed in a JEM 1400 transmission electron microscope (JEOL Co., Tokyo, Japan 2008) at 80 kV and imaged with an 11 Megapixel TEM Morada G2 (EMSIS GmbH, Munster, Germany) camera.

#### 4.4.5. Morphometric Analysis of TEM Images

By comparing the scale bar with the length and width of cell processes, ER, and mitochondria, their median longitudinal (length) and transverse (width) dimensions were calculated by means of ImageJ 1.54i software (National Institutes of Health, Bethesda, MD, USA). We analyzed 10 representative images from one different HaCaT cell each. In each image, we labeled at least 15 cell processes, at least 10 mitochondria, and 10 ER cisternae. Due to their different cross-sections in space, we analyzed only changes in their width. In both cases, length and width was counted from the outermost layer of membranes starting at one pole of the organelle to the outer layer of the membrane at the other pole of the organelle. Length was defined as the dimension along longer axis and for width along the shorter axis. In the case of cellular processes, we considered their length, counting the distance from their origin on the cell membrane, ending at the most distal point of the process. In case of curved processes we have measured their actual length rather than the shortest straight line between two determined points.

#### 4.4.6. Statistical Analysis

Statistical analyses have been performed using the GraphPad Prism version 9.0 for macOS (GraphPad Software, San Diego, CA, USA). The distribution was analyzed with the Kolmogorov–Smirnov test. Normally distributed data were presented with means and standard deviation (SD), whereas the non-parametric data were presented with median and percentiles 25th and 75th (interquartile range). Categorical variables were presented by percentages within each group. Between-group comparisons were carried out with one-way ANOVA test. The results were considered significant for *p* < 0.05.

## 5. Conclusions

PPT is toxic to healthy human keratinocytes even at low concentrations. As demonstrated with its fluorescent derivative (which forms complex with tubulin just like the parent compound), PPT promptly enters the cells, probably via endocytosis. PPT-FL is a useful tool to study intracellular changes associated with PPT treatment. At lower concentrations PPT causes swelling of the ER cisternae under ER- and mitochondrial stress. At higher concentration this leads to massive vacuolization of the cytoplasm with subsequent cell death occurring with longer incubation times. Human keratinocytes display a biphasic response to PPT suggesting the presence of adaptive mechanism(s) requiring further studies.

## Figures and Tables

**Table 1:** Molecular docking and MM/GBSA calculations result for the complexes formed between tubulin and PPT/PPT-FL.

Tubulin Structure Refcode	Ligand	Glide GScore [kcal/mol]	MM/GBSA ΔG Binding [kcal/mol]	Residues Forming Hydrogen Bonds
1SA1	PPT	−8.11491	−61.9107	THR179
	PPT-FL	*	*	*
1TVK	PPT	−5.13944	−47.1387	ASN101, CYS241
	PPT-FL	−3.85146	−51.9734	THR274, ARG282, GLY360
4O2B	PPT	−7.59811	−15.5007	THR274
	PPT-FL	*	*	*
3HKD	PPT	*	*	*
	PPT-FL	*	*	*

* No stable conformation has been found during the molecular docking.

**Table 2:** Detailed information on the crystal structures of the complexes used for the molecular docking.

PDB Refcode	Resolution [Å]	Ligand Present at the Binding Site	Location of the Binding Site
1SA1	4.20	Podophyllotoxin	Between α and β subunits, closer to β
1TVK	2.89	Epothilone	β subunit, on the surface
4O2B	2.30	Colchicine	Between α and β subunits, closer to β
3HKD	3.70	(3Z,5S)-5-benzyl-3-[1-(phenylamino)ethylidene]pyrrolidine-2,4-dione	β subunit, inside

**Table 3:** Matrix plot for root-mean-square deviation (RMSD, Å) analysis. Yellow color for values above 2.0 Å, dark green for values lower than 2.0 Å but higher than 1.0 Å, light green for values less than 1.0 Å.

	1SA1	3HKC	3HKD	3HKE	3N2G	3N2K	3UT5
1SA1	0.186	1.748	2.081	1.579	1.843	0.983	1.125
3HKC	0.874	0.218	1.482	2.210	1.645	0.598	0.874
3HKD	0.596	0.874	0.235	1.764	1.711	0.784	0.986
3HKE	1.417	1.742	1.784	0.342	1.832	0.899	0.763
3N2G	2.081	1.385	1.463	2.145	0.217	0.698	0.984
3N2K	1.573	1.428	1.748	1.125	0.874	0.213	0.892
3UT5	0.421	1.859	1.984	1.854	1.461	1.123	0.329

## Data Availability

The raw data supporting the conclusions of this article will be made available by the authors on request.
